# Experiments Showing the Occurrence of Vegetable Organisms in the Human Blood

**Published:** 1868-07

**Authors:** Joseph G. Richardson

**Affiliations:** Union Springs, Cayuga County, N. Y.


### Experiments Showing the Occurrence of Vegetable Organisms in the Human Blood.

BY JOSEPH G. RICHARDSON, M. D.,
Union Springs, Cayuga County, N. Y.

In the course of my examinations of nearly one thousand specimens of human blood during the past year, I have, in a large proportion of cases, met with the molecular substance denominated by Prof. Salisbury "*zymotosis translucens.*" In severe cases of rheumatism and neuralgia I have found long strings of these transparent granules, and occasionally homogeneous filaments; in the blood of patients afflicted with some other diseases, and of individuals enjoying comparative health, these particles were single, or adherent in rows of two to five or more, such rows often showing a tendency to become branched. They are doubtless identical with the so-called "globulins" of Donné (*Cours de Microscopie,* p. 85, Paris, 1844,) and the "molecular substance" of Griffith and Henfry (*Micrographic Dictionary,* 2d edition, p. 92, London, 1860.) But, in addition to them, I was at first surprised to find in a few instances, that the blood contained in almost every field numerous minute, rounded particles, much more distinct than those above mentioned; not, like them, fading rapidly from view; having an active rotary or erratic motion, and strongly resembling the primary stage of certain infusoria, as seen in solutions of decomposing animal matter. They appeared in cases where the pulse was feeble and intermittent, the blood anæmic, and the powers of life at a low ebb; and diminished in number under tonic treatment, especially the administration of tincture of chloride of iron. It occurred to me that by this property of independent movement we might be able to recognize the existence of independent organisms within the blood, and thus obtain a strong presumptive evidence in favor of Prof. Salisbury's novel theories concerning the vegetable origin of disease; and in order to test the correctness of this surmise the following investigation was undertaken:

*Expt.* 1.—A drop of blood drawn from my own arm was placed upon a slide, and a minute portion of water, which had been standing four days upon some fragments of beef, and which, examined a few minutes before, exhibited multitudes of vibriones, was mixed with it, and the whole covered with a thin glass. Upon adjusting it under the microscope, the vibriones were found to be moving, some rapidly, some slowly, and some only as borne by the currents among the blood-corpuscles, apparently unaffected by the change of world which they had undergone. By arranging a filament of thread from the reservoir upon the growing slide, so as to supply the loss of fluid by evaporation, I was able to watch their progress, at short intervals, for about nine hours, which was, as far as I could judge, uninterrupted toward the formation of vibratile filaments resembling the early stages of development in the so-called *Leptothrix Baccalis*, found so abundantly in the tartar on the teeth. One particular filament, which was carefully watched, and of which drawings were made from time to time, whose movement, when first observed, was very active and constant, grew from a length of about $\frac{1}{1500}$th of an inch to that of about the $\frac{1}{1000}$th of an inch in eight hours, to that of about $\frac{1}{800}$th of an inch in seventeen hours; and at the end of twenty three hours, when the experiment was interrupted, had attained a length of about $\frac{1}{500}$th of an inch. The process of development seemed to be accompanied with a disposition to bend sharply at intervals of perhaps $\frac{1}{2000}$th of an inch, and shoot forth from the salient angle a branch equal in size to the parent trunk. As the organism increased in length, its movement diminished in rapidity, until towards the close of the experiment it nearly or quite ceased; its breadth continued the same throughout, and appeared to be about $\frac{1}{20000}$th of an inch.

According to the conclusions of Prof. J. Wyman, in his paper on the existence of living organisms in heated water,* neither vibrious nor bacteriums appeared (in water containing beef-juice) if the boiling was prolonged beyond the period of five hours; and the Professor quotes Pasteur† to the effect that the spores of some kinds of cryptogams (even those most salamander-like, he appears to mean) perish at a dry heat of 266° F., so that the slides and covers used in the following experiment, being, after thorough cleansing, burnt off in the flame of an alcohol lamp, may probably be considered free from any such impurity.

*Expt.* 2.—January 6, 1868, at 8½ P. M., two hours after a slight supper, I drank a fluid ounce of water, which, having stood upon some fragments of beef for two days, contained, as counted under the microscope, on an average (the mean of ten enumerations) about 14 vibriones and bacteria to each square $\frac{1}{1000}$th of an inch; a drop (or minim) being spread out under a thin glass one inch square, so that the f℥j included, in round numbers, 7,000,000,000 of living organisms. This compound, although sufficiently repugnant to the palate, had no nauseating effect upon the stomach beyond that fairly attributable to mental disgust, and probably possessed no higher aroma than a professed gourmand used to enjoy in the saddle of venison which had garnished his larder until it acquired the true game flavor. Half an hour after the imbibition of the mixture, a drop of blood drawn from my arm, and examined on a slide simply wiped clean, showed, on rigid scrutiny during another half hour, but a single moving molecule. At 9½ P. M. a glass and cover heated far beyond the limit above given as compatible with organic life, and scrupulously protected from exposure to deposits from the atmosphere, were used for the examination of another drop of blood, in which four molecules in active motion, precisely resembling that of infusoria seen in Expt. 1st, were visible. A drop drawn at 10 P. M., and examined between a glass and cover prepared with the same precautions, exhibited six specimens of moving bodies; while in a drop drawn at 10½

* See this Journal, p. 283, January, 1868.

† Am. Journ. Sc. and Arts, p. 164, from Ann. de Sci. Nat., t. xvi, p. 81, 1861.

P. M., only two were detected during a careful search of half an hour's duration.

*Expt.* 3.—At 7 P. M., January 7, 1868, four hours after dinner, I swallowed four fluid ounces of water which had been standing some seventy hours upon fragments of beef, and which, according to the data of experiment 2d, contained at least 27,000,000,000 living organisms. As this test was intended to be as far as possible a crucial one, at 8 o'clock I prepared a slide and cover in the following manner: after washing them thoroughly and drying them on a clean cotton cloth I applied a drop of strong hydrochloric acid to the middle of the slide and laid upon it the glass cover, taking care that by suitable pressure the acid was evenly distributed between the surfaces; raising the cover after about a minute I held it by means of forceps in the flame of an alcohol lamp until all the acid was volatilized and then placed it carefully under a small bell glass—the slide itself was similarly treated, and when both were quite cool a drop of blood (obtained from an incision made through integument painted with tr. ferri chlor.) was touched to the slide which was quickly transferred beneath the bell glass applied to the glass cover, and the whole reversed and placed upon the microscope stage. The lenses being adjusted, I found the blood remarkably full of moving particles precisely resembling to my eye specimens of vibrio bacillus; these were so abundant that I counted twelve in about as many minutes, and at one time three were visible in the same field. At a quarter before nine another drop of blood drawn from a new incision near the last was examined between a slide and cover prepared exactly as the previous one, and with the same result except that the revolving particles were fewer in number, only four being observed whose motion was unmistakable. At half-past nine another drop from the second incision re-opened was examined between a slide and cover that had been simply heated without the application of acid, and on careful scrutiny for about half an hour only revealed three moving molecules. In examining for these I found a satisfactory method, after discovering one which changed its place under the lowest eye-piece, was to put on that containing the cobweb micrometer, by which at least mistakes proceeding from oscillation of the head or vibration of the instrument were readily corrected.

But such investigations being made with only a one-eighth inch objective, and the lowest eye-piece of a Powell and Lealand's instrument, could not furnish positive proof that these moving particles were not merely inorganic matter undergoing molecular movement, or that if organized they were not the primary constituents or disintegrating residuum of white blood-corpuscles, and I therefore obtained from Mr. Wm. Wales an "Immersion" lens, having nominally but one-twenty-fifth inch focal length, for the purpose of verifying and extending my conclusions. This glass affords a power of about eleven hundred diameters with very clear definition, and after some preliminary study of the organisms in decomposing beef-juice, I made with its aid the following researches:

*Expt.* 4.—At 7.45 P. M., May 17, 1868, I drank four fluid ounces of water similar to that employed in the preceding investigations, and containing multitudes of bacteria. At a quarter past eight I examined a drop of blood drawn with a cataract needle from the tip of my finger, and confined between a slide and cover cleaned with strong hydrochloric acid as above described; under the field of the one-twenty-fifth inch glass, the interspaces between the rows of blood-corpuscles were found to contain multitudes of apparently spherical molecules in rapid and erratic motion—but so very minute as to readily escape notice even with this high power, except under the closest scrutiny; in the course of half an hour not less than one hundred were observed. At nine P. M., another drop of blood examined with the same precautions exhibited in addition to these minute particles, other bodies less active in their movements, of much greater magnitude, and which under an amplification of eleven hundred diameters, appeared precisely similar to the bacteria I had been studying a few hours before in the identical decomposing beef-juice imbibed. Five of them were thus enlarged sufficiently to exhibit an unmistakable organized structure totally different from their associated aggregations of Beale's "germinal matter." (Plate xxvii, Fig. 208, *Microscope in Practical Medicine.)* Three of these bacteria were each about $\frac{1}{12000}$th of an inch in length and $\frac{1}{25000}$th of an inch in width, very distinctly constricted in the middle; a fourth was obviously composed of four and a fifth of six joints, arranged in a straight line, whose motion was of that peculiar waving character so universal among the Oscillatoriaceæ—the last two were most clearly visible when they happened to lie vertically to the surface of the glass, and would probably escape observation under the one-eighth inch, except in that position, and be therefore mistaken for simple globular bodies, although in several cases I detected in the second and third experiments a shadowy elongation of one diameter in the revolving molecules then observed.

In view of the statement of M. Davaine to the French Academy of Medicine (*Medical News and Library*, vol. xxvi, p. 28,) asserting a close connection between the appearance of bacteria in the blood and the occurrence of carbuncular disease, it is worthy of remark that neither at, nor subsequent to, either of the three occasions in which I thus impregnated my blood with infusoria, were there any symptoms of carbunculous or other inflammatory malady. The only disturbances of the economy observed were headache, furred tongue, dryness of the throat, and slight diarrhœa, which all passed off in a day or two, the offending organisms being apparently soon eliminated by the various outlets for effete or noxious materials; even these deviations from health may have been accidents, the results of other causes.

Although I am well aware that the plans adopted do not preclude the possibility of error through the introduction of living organisms into the blood after it has left the walls of its vessels, yet I think most candid inquirers will admit that the fact that an increased number of moving particles were visible after an increased dose of vibriones (contained in the draught above mentioned *whose swarming population exceeded more than twenty times the sum total of every man, woman and child, who walks upon our earth,*) and at the same time *in spite* of increased precautions which the most stubborn skeptic must acknowledge would have a tendency to diminish the chances of deception, goes far to prove that multitudes, probably millions, of infusoria, thus entering the stomach, find their way into the blood in a few hours, and, retaining their independent vitality, circulate with that vital fluid through the minutest ramifications of the arteries, and penetrate to every portion of the human system. And if this be true, how strong becomes the presumption that there are other plants more deleterious in their growth or more poisonous in their nature, which also thrive under certain circumstances within the blood, and each constitute the essence, the real *contagium*, of some so-called zymotic disease, as, for example, diphtheria and scarlet fever, small-pox and measles, as declared long ago by Prof. Salisbury, of Cleveland, and recently by Prof. Hallier, of Jena.—*Am. Jour. of Medical Sciences for July*, 1868.

